# In-situ electrochemical impedance analysis of a commercial SOFC stack fueled by real wood gas

**DOI:** 10.1016/j.heliyon.2024.e32509

**Published:** 2024-06-06

**Authors:** Federica Torrigino, Fabian Grimm, Jürgen Karl, Katharina Herkendell

**Affiliations:** Institute of Energy Process Engineering, Friedrich-Alexander-Universität Erlangen-Nürnberg, Fürther Str. 244f, 90429, Nuremberg, Germany

**Keywords:** Solid oxide fuel cell (SOFC), Biomass, Wood gas, Electrochemical impedance spectroscopy (EIS), Distribution of relaxation times (DRT), Distributed power generation

## Abstract

The combination of solid oxide fuel cells (SOFCs) and wood gasification has the potential to significantly increase renewable electricity production and decrease emissions. Depending on the quality of the wood gas, degradation processes have a significant impact on the reliability and lifetime of the SOFC. Using electrochemical impedance spectroscopy (EIS) and subsequent distribution of relaxation times (DRT) analysis, the impact on the degradation of coupling wood gasification with a commercial SOFC stack is determined in this study. The thermal behavior of the SOFC stack under various operating conditions, as well as various synthetic wood gas mixtures classified by their hydrogen-to-carbon (H/C) ratio, was assessed. The decrease in the H/C ratio from 8 to 1, observed during syngas and real wood gas operation, leads to a rightward shift in the Nyquist plots, suggesting an increase in the SOFC stack's impedance. Correlations between variations in the H/C ratio and their effects on anodic electrooxidation, ionic conduction, gas transport, and diffusion were identified using DRT analysis to interpret the EIS results. By incorporating an upstream desulfurization system and ensuring an H/C ratio greater than 2, the coupling of biomass gasification with the SOFC stack was stable to degradation issues.

## 1 Introduction

The European Green Deal (EGD) intends to reduce greenhouse gas emissions by at least 50 % in Europe by 2030. To achieve this goal, a transition from the use of fossil fuels toward renewable fuels is required. In the field of heat and power generation, many environmentally friendly alternatives for reaching carbon neutrality have been discussed [[1], [2], [3]]. Among these, fuel cell technology stands out due to its potential to convert the chemical energy of fuel into electricity via an electrochemical process with high efficiency and low carbon emissions [[4], [5], [6]].

Because natural gas (NG) is heavily utilized for heat and power generation, switching to a renewable gas source is crucial to addressing the environmental objectives set for 2030. Gasification of solid biomass to generate syngas is a possible carbon-neutral approach for accomplishing this ambitious goal. In this scenario, combined heat and power (CHP) solutions might make a significant impact in balancing and improving the system's effectiveness, and it appears to be the best strategy for optimal syngas utilization [3,[7], [8], [9]]. Another appealing idea for power generation in recent years has been to switch out conventional internal combustion engines (ICE) with a SOFC fueled by syngas, which would enhance electrical efficiency by more than 40 % [[10], [11], [12], [13]]. Because biomass gasification is commonly carried out at high temperatures (up to 900 °C), gasification and SOFC integration are considerably easier than with other forms of high-temperature fuel cells [14,15]. Since a single SOFC has a low power voltage (i.e. well below 1 V), for technical applications more single cells are electrically connected in series to form a so-called “stack”. Several types of SOFC configurations have been proposed. The differences among these designs are the shape of each cell, the method of connecting each cell, and the flow of fuel and oxidants through their channels [[16], [17], [18], [19]]. This work utilizes an open SOFC stack design, where the unused reactant gas is burned off in an afterburner chamber. Several issues remain to be addressed for SOFCs in order to be an interesting option for commercialization. Aside from economic viability, the biggest hurdle is achieving long-term degradation-free operation of SOFCs. Adverse operating conditions or fluctuating gas quality can lead to various degradation effects such as nickel oxidation and nickel agglomeration at the anode. The described degradation effects lead to a greatly reduced SOFC lifetime and performance loss [16,[20], [21], [22], [23]].

The feedstock utilized for the gasification has a significant impact on the quality of the syngas generated. Due to its high calorific value and relatively low contaminant level, woody biomass is one of the most frequently used types of biomass for SOFC cogeneration applications and was utilized in this study [21,24,25]*.* The resulting syngas, also known as wood gas, is a gas mixture mostly composed of methane and carbon dioxide as well as contaminants including sulfur compounds and heavier hydrocarbons (tars) [8,24]. Although SOFCs are more resistant to tars than traditional engines, the presence of tars causes carbon deposition at SOFC electrodes, reducing nickel catalytic activity and is considered one of the main concerns for achieving high performance and ensuring safe operation of SOFCs [[26], [27], [28]]. The behaviour of tars on SOFC anodes has attracted much attention recently [[29], [30], [31], [32], [33], [34]]. Because the real tar in gasification result is a complicated mixture, most researchers employ a single tar component as a model on SOFC to assess its direct influence on anodes [31,[35], [36], [37], [38]]. Sulfur compounds, in addition to tars, are another barrier to a stable, long-term, and high-performance SOFC operation. Hydrogen sulfide (H_2_S), an inorganic sulfur molecule, and other organic sulfur components such as mercaptans and thiophenes could instantly deactivate the electrochemical activity of SOFCs at concentrations of several ppm [[39], [40], [41], [42]]. Syngas from the gasification of biomass contains both, inorganic and organic sulfur components at significant levels [43]. Traditional electrochemical characterization techniques for analyzing these degradation effects include polarization curve measurement and EIS. Impedance spectroscopy, specifically, enables real-time and non-destructive analysis of systems. It facilitates the assessment of performance parameters and the identification of degradation processes, which are regarded as significant challenges in the field [[44], [45], [46]].

Recent studies have investigated the impact of real biosyngas on SOFC performance and have highlighted the importance of optimizing operating conditions for efficient and degradation-free operation. Li et al. underscored the significance of understanding the interaction between gas components or contaminants in biomass gasification-coupled SOFC systems. They highlighted that under unfavorable gas or thermal conditions, micro-/macrostructural changes, delamination, or even cell fractures could occur, leading to performance reduction or irreversible cell damage. Therefore, identifying safe operating conditions and developing advanced cell materials are critical aspects of biomass-coupled SOFC systems [25]. Pongratz et al. demonstrated the feasibility of operating SOFCs with real product gas containing tars from steam gasification. They emphasized the importance of reducing heavy tars to prevent structural degradation of the anodes and performance losses [13]. Similarly, Subotic et al. conducted a comprehensive analysis to determine preferable operating SOFC conditions for integration with real steam gasifiers of biomass. Their study highlighted the significance of high steam levels and current loads in reducing the risk of carbon deposition, a significant concern for long-term operation [21]. Moreover, both studies integrated an analysis of EIS spectra with other characterization techniques to obtain a more in depth comprehension of the SOFC performance and degradation mechanisms. Inac et al. studied the impact of biosyngas composition on SOFC anodes using C–H–O ternary diagrams to evaluate the thermodynamics of carbon deposition. Their impedance measurements indicated that Ni-based anodes could maintain reasonable performance with biosyngas compositions, showing no impact up to a certain limit of H_2_S (9 ppm) [47].

The initial experimental phase focused on variating key parameters, including temperature (T) and volumetric flow rate of inlet gas (${\dot{V}}_{\text{in}})$. In the second phase, different types of fuels were used, including reference gases, synthetic wood gas mixtures, and eventually real wood gas. EIS characterization provides non-destructive online diagnostics to reveal essential operational parameters and degradation processes. The in-situ EIS spectra were then analyzed using the DRT method in a post-process analysis. Despite overlapping physical processes in the Nyquist diagram, the DRT allows them to be distinguished by their relaxation time, τ, which carries information on the rate at which the process occurs. As a result, the number of peaks in the DRT analysis is proportional to the number of processes having an impact on the cell resistance. DRT analysis extracts insights from the Nyquist diagram, assuming a model similar to the Randles circuit with an unspecified number of poles. It often utilizes Tikhonov regularization for estimating relaxation time distributions. Similarly, fitting the Randles ECM directly from EIS data can provide DRT in a closed form. While DRT analysis offers information extraction without prior ECM modeling, it's important to acknowledge its assumptions and alternative methodologies. Furthermore, the width of the peaks is an indicator of process homogeneity [[48], [49], [50], [51], [52]]. It is important to note, that the primary objective of the work was to perform a coupling with a real gasification system and obtain a more detailed interpretation via EIS to acquire a more comprehensive interpretation using the DRT approach in contrast to a more general investigation utilizing, for example voltage-current curves. While previous studies [7,9,13,21,24,30,31,33,34,37,39,[53], [54], [55]] have investigated the impact of real wood gas on SOFC performance using current-voltage curves, post-mortem techniques or EIS, the present work provides a unique contribution by applying the DRT approach to interpretate the EIS spectra. With the application of DRT analysis approach, it is possible to extract the most dominant time constants or frequencies for the processes involved, providing deeper insights into the electrochemical processes within the SOFC stack. The interpretation of the DRT analysis is based on previous literature and permits the identification of specific peak regions [[49], [50], [51],^56^,^57^]. This method yields a more pragmatic approach; consequently, the presented DRT analysis results may appear more generic.

**Table:** 

**Abbreviations**	
CHP	Combined Heat and Power
CPOx	Catalytic Partial Oxidation
DRT	Distribution of Relaxation Time
ECM	Equivalent Circuit Model
EIS	Electrochemical Impedance Spectroscopy
f	Frequency
FRA	Frequency Response Analyzer
H/C ratio	Hydrogen to Carbon ratio
H_2_S	Hydrogen Sulfide
i_L_	Load Current (DC-DC converter)
KK relations	Kramers-Kronig relations
LHV	Lower Heating Value
NG	Natural Gas
OCV	Open Circuit Voltage
SOFC	Solid Oxide Fuel Cell
SPE	Solid Phase Extraction
T	Temperature
U/i curve	Voltage/current curve
${\dot{V}}_{i}$	Volumetric flow rate of the gas component *i*
${\dot{V}}_{\text{in}}$	Volumetric flow rate of the anodic inlet gas
λ_air_	Air-fuel equivalence ratio
λ_reg_	Regularization parameter
τ	Relaxation Time

## 2 Experimental

### 2.1 Experimental setup

In this study, the SOFC stack sourced from the Galileo 1000 N SOFC-based heating system, which is produced by Hexis AG has been used. The SOFC stack possesses an active cell area of 100 cm^2^ and generates an electric power output of 1 kW_el_. The system consists of 62 electrolyte-supported individual cells (ESCs) separated by metallic interconnectors (MICs) and employs Ni-CGO as the anodic material and LSM-8YSZ as the cathodic material. Even though hydrogen is considered the standard fuel for SOFCs in the literature [5,14,16], the Hexis-stack, as it is designed for house heat and energy supply, utilizes pre-reformed NG. Four air inlet lines supply preheated air to the cathode side of the cells. The post-combustion chamber encloses the fuel cell module, reducing seal demands and simplifying the thermal management system [58,59]. The fuel cell module utilizes catalytic partial oxidation (CPOx) to sub-stoichiometrically pre-reform natural gas with an air-fuel equivalence ratio (λ_air_) of 0.27. For λ_air_ > 0.26, the standard CPOx unit temperature is reported in the literature to be above 750 °C [60]. The temperatures of the unit in our experimental setup were in this range at the start of the experimental sets but decreased to between 300 and 400 °C by the end of the sets. The observed phenomenon may be attributable to a decline in the catalytic activity of the nickel catalyst in the CPOx unit over time. Multiple thermocouples have been placed at the top, middle, and base of the stack to regulate its operating temperature. A programmable logic controller (PLC) system designed for industrial applications by B&R Industrie Elektronik GmbH was successfully programmed and implemented in order to fully automate the SOFC stack. The system's automation allows for precise control of operating parameters, such as temperature and volumetric flow rate within the SOFC stack, thereby ensuring strict adherence to all safety protocols.

The gas mixture analysis at the stack inlet was performed using a gas analyzer (AO2020 Caldos 25 with Uras 26, ABB). Within this work the SOFC stack has been coupled to an in-house developed allothermal gasifier (operating temperature of 800 °C at 0.6 bar) with a volumetric flow rate of 2–4 l min^−1^ real wood gas. A pre-cleaning unit composed of two scrubbing bottles and an active carbon filter was used to remove part of the tars and sulfur components from the real wood gas before entering the SOFC stack. The composition of the purified real wood gas is reported in Table 1. The presence of H_2_S in the wood gas was determined using hydrogen sulfide detector tubes in different scales (no. 4LL, GASTECH).

**Table 1:** Gas composition of the lab-scale gasifier with the corresponding volumetric fractions values.

Gas compound [−]	Volumetric Fraction [−]
CO	0.18
H_2_	0.40
CO_2_	0.24
N_2_	0.12
CH_4_	0.06

In order to precisely measure the quantity of tars in the wood gas produced by the laboratory-scale gasifier and subsequently comprehend the impact of these tars on the SOFC system, the wood gas originating from the allothermal gasifier was analyzed concerning its impurities using the solid-phase extraction (SPE).

**Fig. 1:**
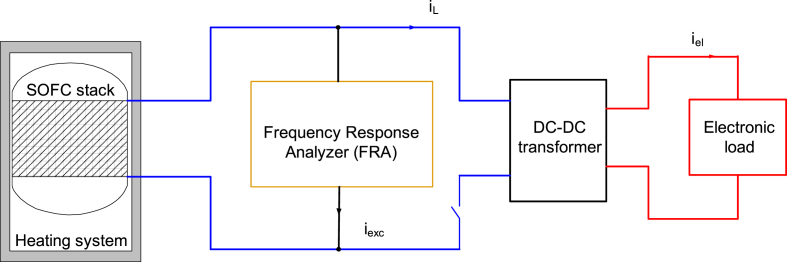
Schematic illustration of the experimental setup and current flows consisting of the SOFC-stack integrated into the Galileo 1000 N heating system. Additionally, it shows the FRA device responsible for regulating the current excitation (i_exc_), the DC-DC converter controlling the external load (i_L_), and the electronic load with its current (i_el_).

The electrochemical analysis was performed using a galvanostatic frequency response analyzer (FRA) developed by NOVUM engineering GmbH. The galvanostatic measurement method is used in situations where the impedance being measured is very low, which is the case in this scenario. In this experimental setup, the system is subjected to an alternating current with a predetermined amplitude, and the resulting voltage response is measured. To simulate a consumer an electronic load (EL9000-HP, EA Elektro-Automatik) was used. Prior EIS measurements on the SOFC stack under varying consumer-applied external loads revealed significant signal noises, rendering the spectra unreliable. The observed noises were most likely caused by transient elements in the load's regulated current. The transient components behave like the FRA's current excitation and generate an AC voltage across the stack impedance. This AC voltage is detected by the FRA and falsifies the impedance spectrum, as the transient current components of the load current cannot be determined. To overcome this issue and to perform a reliable electrochemical analysis, the electrical circuit was split using a DC-DC converter (NOVUM engineering GmbH) in Fig. 1. For further information on the circuit's layout, please refer to the supplementary material.

With this arrangement, the voltage drop across the SOFC stack and its impedance are determined by the constant load current of the DC-DC converter (i_L_) and the FRA's excitation current (i_exc_). The voltage drop remains unaffected by the presence of the electronic load (iel), thereby eliminating any potential signal noise that could interfere with the accuracy of the EIS measurements. This concept was demonstrated to be crucial, but it also imposed a technical limitation to the power of the stack, which was limited to a maximum power of 240 W_el_.

### 2.2 Experimental sets

Two experimental sets were carried out to evaluate the effects of temperature increase and variation of ${\dot{V}}_{\text{in}}$, as well as to assess the impact of coupling the lab-scale wood gasification to the system, as shown in Table 2. The first section of experiments comprised a cell temperature variation of 800 °C, 820 °C, and 850 °C with a constant pre-reformed NG supply of 2.5 l min^−1^ (tests 1a-c). In the second test section, ${\dot{V}}_{\text{in}}$ was varied at a constant cell temperature of 820 °C between 2.1 l min^−1^, 2.5 l min^−1^, and 2.9 l min^−1^ (tests 2a-c). All experiments in this experimental set were carried out at open-circuit voltage (OCV) conditions without the application of any external load.

**Table 2:** Overview of both experimental sets with their relevant operating parameters.

	Test no.	Fuel [−]	T [°C]	LHV [kWh_th_ m^−^³]	${\dot{V}}_{\mathrm{in}}$ [l min^−1^]	i _L_ [mA cm^−2^]
**Experimental set 1**	1a	Pre-reformed NG	800	10.15	2.5	–
	1b	Pre-reformed NG	820	10.15	2.5	–
	1c	Pre-reformed NG	850	10.15	2.5	–
	2a	Pre-reformed NG	820	10.15	2.1	–
	2b	Pre-reformed NG	820	10.15	2.5	–
	2c	Pre-reformed NG	820	10.15	2.9	–
**Experimental set 2**	3a	Pre-reformed NG	820	10.15	2.5	10
	3b	Hydrogen	820	2.99	9.8	10
	4a	Synthetic wood gas	820	2.00	11.76	10
	4b	Synthetic wood gas	820	2.00	12.40	10
	4c	Synthetic wood gas	820	2.00	13.67	
	5a	Real wood gas (+H_2_)	820	1.59	9.8	10
	5b	Real wood gas (+H_2_)	820	1.78	11.8	10

The second experimental set was divided into three sections according to the type of fuel gas used to power the stack: reference gas, syngas, and wood gas. All tests were performed at a constant temperature of 820 °C and a load current of the DC-DC converter (i_L_) of 10 mA cm^−2^.

During the first section of the second experimental set (tests 3a-b) the SOFC stack was fed with pure hydrogen and pre-reformed NG respectively to establish a benchmark for the following experiments. In the subsequent section, a preliminary investigation (tests 4a-c) using three different syngas compositions as fuel gas was performed. The purpose of this section was to predict the critical operational parameters of the SOFC system and its precise thermal behavior by implementing syngas experiments designed to simulate the operation with real wood gas. The last part of the experimental set involved the use of wood gas to fuel the SOFC stack (tests 5a-b). This section aimed to examine the behavior of the SOFC stack when coupled to a lab-scale gasifier. In accordance with the stack manufacturer, Hexis AG, the SOFC stack must be operated between 1.27 kW_th_ and 3 kW_th_ for safety reasons. For the testing of syngas, an operational average heating power of 2 kW_th_ was selected and the software FactSage [61] was utilized to calculate the volumetric flow rates of all gas mixtures in accordance with this value. The gaseous mixture entering the system was primarily composed of carbon monoxide (CO) and hydrogen (H_2_) and methane (CH_4_), carbon dioxide (CO_2_), and nitrogen (N_2_) in small amounts. To ensure the safe operation of the stack, an additional quantity of hydrogen (7.8 l min^−1^) was introduced into the inlet gas during real gas operation. The lab-scale gasifier's operational constraints prevented it from achieving the necessary boundary safety conditions. Table 3 presents the volumetric flow rate and corresponding heating value obtained.

**Table 3:** Overview of the volumetric flow rates for each gas component and the resulting heating value of the inlet anodic gas mixtures used for the second experimental set.

Test no.	Fuel	${\dot{V}}_{H_{2}}$	${\dot{V}}_{{CH}_{4}}$	${\dot{V}}_{CO}$	${\dot{V}}_{{CO}_{2}}$	${\dot{V}}_{N_{2}}$	${\dot{V}}_{\mathrm{in}}$	LHV
	[−]	[l min^−1^]	[l min^−1^]	[l min^−1^]	[l min^−1^]	[l min^−1^]	[l min^−1^]	[kWh_th_ m^−^³]
3a	Pre-reformed NG	0	2.5	0	1	1	2.5	10.15
3b	Hydrogen	9.8	0	0	0	0	9.8	2.99
4a	Synthetic wood gas	9.71	0.21	0.62	0.82	0.41	11.77	2.00
4b	Synthetic wood gas	8.30	0.41	1.23	1.64	0.82	12.40	2.00
4c	Synthetic wood gas	5.47	0.82	2.46	3.28	1.64	13.67	2.00
5a	Real wood gas (+H_2_)	0.6 (+7.8)	0.1	0.1	0.38	0.82	9.80	1.59
5b	Real wood gas (+H_2_)	1.2 (+7.8)	0.02	0.02	0.76	1.64	11.80	1.78

In order to conduct a comprehensive analysis of the results, the gases and mixtures utilized in the study were further categorized according to their H/C ratio as can be seen in Table 4. This ratio relates the molar hydrogen content (H-content) to the molar carbon content (C-content) of gas mixtures, where the molar masses of hydrogen and carbon have been assumed to be 1.01 g mol^−1^ and 12.01 g mol^−1^, respectively.

**Table 4:** Overview of the molar H- and C-content and resulting H/C ratios for each test conducted in the second experimental set.

Test no.	Fuel	Atomic H-content	Atomic C-content	H/C ratio
	[−]	[mol min^−1^]	[mol min^−1^]	[−]
3a	Pre-reformed NG	0.44	0.11	4
3b	Hydrogen	0.22	–	∞
4a	Synthetic wood gas	0.25	0.07	3
4b	Synthetic wood gas	0.26	0.15	2
4c	Synthetic wood gas	0.27	0.29	1
5a	Real wood gas (+H_2_)	0.21	0.03	8
5b	Real wood gas (+H_2_)	0.24	0.05	5

During tests 3a and 3b, the SOFC stack was fed with hydrogen and pre-reformed NG with an H/C ratio of “infinitely large” and 4, respectively. The tests 4a-c involved the utilization of three distinct mixtures containing varying proportions of synthetic wood gas with H/C ratios of 3, 2, and 1. In tests 5a and 5b real wood gas, the volumetric flow rate of wood gas was changed from 2 l min^−1^ to 4 l min^−1^ with H/C ratios of 8 and 5, respectively.

### 2.3 Electrochemical characterization

The EIS and subsequent DRT analysis have been used to characterize each experimental set. The impedance of a system can be determined by applying a sinusoidal current and analyzing the resulting voltage response. The impedance's real and imaginary parts can be visually depicted through the utilization of either the Nyquist or Bode plot [44,46,49]. Due to the fact that each of the diagram's semicircles corresponds to a physical process, the Nyquist plot permits the identification of processes occurring within a SOFC such as diffusion, charge transfer, and electron transfer [49,56]. At very high frequencies (f > 1 kHz), the phase shift is zero, resulting in the convergence of the curve with the real axis at the ohmic resistance (R_o_) mainly determined by the resistance of the electrolyte. At lower frequencies (f < 1 Hz), the system approaches another convergence point, resulting in R_ASR_, which is the sum of all polarization processes at a given operating point. The difference between total impedance and ohmic resistance is the polarization impedance (R_pol_), which describes the polarization behavior of the system [44,46,56,57,62]. It's worth noting that an impedance can only be specified for a system that meets the linearity, causality, and time-invariance requirements. If one of these requirements is not met, no meaningful information can be extracted from the measurement [44,46,51,62]. The Kramers-Kronig (KK) relations are commonly used to evaluate the fulfillment of these criteria and subsequently validate the accuracy of the impedance spectrum [63,64].

The interpretation of impedance spectra presents considerable challenges. Physical models of all occurring processes, such as ECM, are required to achieve this goal. However, due to the fact that different equivalent circuits can produce the same response, the use of ECM can be confusing if it is not performed in detail and physical properties are not taken into account [50,52,65,66]. Absent these conditions, processes will overlap and the signal resulting from the fitting model will appear as a single semicircle on the Nyquist diagram. As a method for circumventing this problem, DRT analysis is gaining popularity among researchers. The DRT analysis enables the differentiation of physically similar processes based on their relaxation time, which may overlap in the Nyquist diagram [50,67]. Thus, the relaxation method provides a wealth of information from the Nyquist diagram without the need to model it beforehand using an electrical equivalent circuit. This analysis exploits the fact that in an electrochemical system, each physical process is defined by a time constant called the relaxation time, τ, which contains information about the rate at which the process occurs. Thus, the number of peaks in the DRT analysis corresponds to the number of occurring processes. The peak frequency of the peaks describes the velocity of the respective processes. Furthermore, the width of the peaks is an indicator of process homogeneity [[49], [50], [51], [52]].

In this work, each EIS measurement performed on the SOFC stack was repeated three times, increasing the excitation amplitude from 100 mA to 180 mA within a fixed frequency range (0.1 Hz - 10 kHz). Firstly, the resulting Nyquist plots of the measurements were validated by the KK relations using the LinKK tool. The LinKK tool, developed by the Karlsruhe Institute of Technology (KIT), offers an automated solution for determining the optimal number of RC-elements required to align impedance spectra. This tool is designed for impedance analysis, involving a series connection of an ohmic resistor and multiple RC-elements. It effectively distributes the time constants logarithmically across the frequency range of the impedance spectrum being tested, ensuring a precise representation of the EIS data. The test result displays residuals, indicating the discrepancy between the measured values and an ideal KK spectrum at various frequencies [63,64,68]. Subsequently, the measurement that showed the lowest deviation (<1 %) from the fitting was selected.

In the next step, this validated measurement was analyzed by a freeware DRT tool embedded in a MATLAB environment from Ciucci et al. [69] adjusted for our data range, and the DRT spectrum was then created. The analysis of the spectra is heavily influenced by the technical limitations of the analytical equipment (max. frequency range of the FRA 10 kHz). In this case, it is hard to estimate ohmic and polarization resistance contributions which normally are visible at high-frequency ranges (f > 100 kHz) [49,56,57]. The main challenge for the DRT analysis is the selection of the regulation parameter λ_reg_. For the DRT evaluation, the parameters recommended by Ciucci et al. [52,65,69] were chosen. This parameter adjusts the filtering of the measured data and is a very important step to avoid false peaks in the analysis. There is no strict set of criteria for the selection of λ_reg_. According to Sonn et al. [66], the regulation parameter was gradually decreased starting from larger values (λ_reg_ = 10^−2^) until the DRT spectrum no longer showed systematic deviations. This approach resulted in a λ_reg_ between 10^−5^ and 10^−8^. The DRT spectrum of a SOFC system is typically partitioned into distinct regions that correspond to the various physical processes taking place within the cell, as documented in the literature [48,49]. The software OriginLab (OriginLab Corporation) is utilized to integrate the area encompassed by each peak of DRT in a given region, and subsequently reported to identify the overall processes as well as those specific to each region.

## 3 Results and discussion

### 3.1 Wood gas analysis

**Fig. 2:**
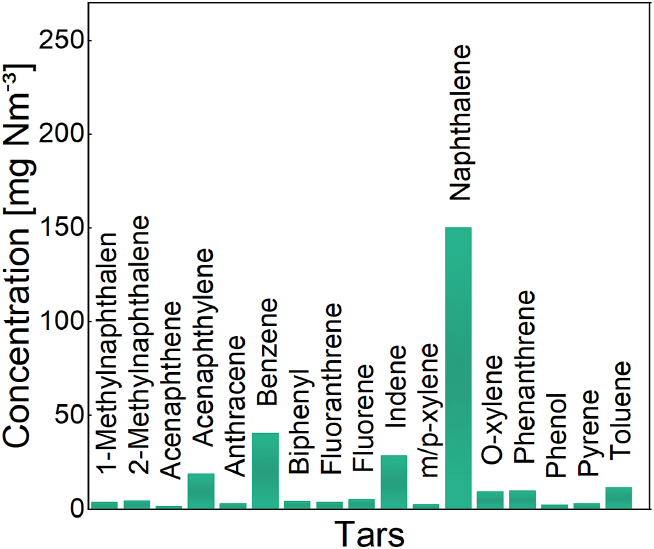
Tar concentration of the real wood gas produced by a lab-scale allothermal gasifier designed by our institute operating at 800 °C, pressure of 0.6 bar and with an outlet volumetric flow rate of 2 up to 4 l min^−1^.

The H_2_S detector tubes revealed the absence of H_2_S in the wood gas entering the SOFC stack, implying the efficient operation of the pre-cleaning unit. Notably, in this instance, the sample extraction point was located after the cleaning unit and prior to the stack. The results of the SPE, shown in Fig. 2, indicate that the predominant tar compounds present in the wood gas are naphthalene (150 mg Nm^−^³), benzene (44.7 mg Nm^−^³), indene (28.5 mg Nm^−^³), acenaphthylene (19 mg Nm^−^³), and toluene (16.5 mg Nm^−^³).

Thus, the gas analysis shows that, on one hand, the specifically implemented gas cleaning unit has achieved an effective pre-cleaning, but that, on the other hand, a small effect of the tar on the SOFC stack behavior should be expected.

### 3.2 Interpretation of EIS spectra

**Fig. 3:**
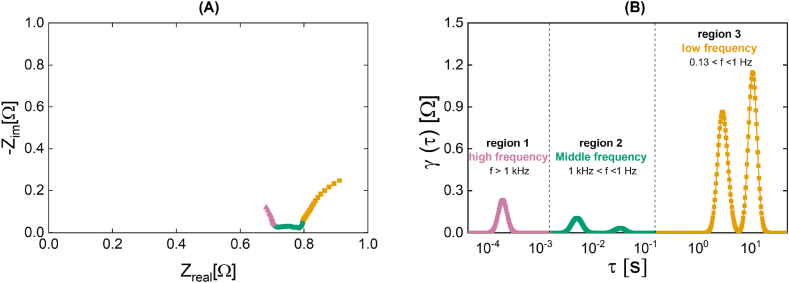
Nyquist **(A)** and DRT **(C)** plots of the SOFC stack at open-circuit voltage (OCV) under standard conditions (820 °C and 2.5 l min^−1^) using NG as fuel. These plots serve as example for the method used for the region identification from the Nyquist to the DRT plot. The high frequency region (f > 1 kHz) of the Nyquist plot is associated to region 1 (pink), the middle frequency region (1 kHz < f < 1 Hz) to region 2 (green), and the low frequency region (0.13 Hz < f < 1 Hz) to region 3 (orange). (For interpretation of the references to colour in this figure legend, the reader is referred to the Web version of this article).

The DRT analysis method was used to extract valuable information and insights from the Nyquist diagram in lieu of constructing an ECM. The LinKK validation tool confirmed the accuracy of spectra below 4 kHz. As a result, data beyond this frequency range, specifically frequencies between 10 Hz and 4 kHz, were excluded when analyzing the EIS spectra. The peak areas are associated to different process occurring within the stack. Fig. 3 explains the evaluation method used in this study by depicting the Nyquist plot (Fig. 3 (A)) and the corresponding DRT plot (Fig. 3 (B)) derived from a standard measurement of the investigated SOFC system using pre-reformed NG under OCV load conditions.

Moreover, the figure depicts the respective region subdivisions. Region 1 (pink) is characterized by the high frequency range in the Nyquist plot and is associated with rapid electrochemical processes occurring at the electrodes, such as the anodic electrooxidation reaction and the ionic conduction of the electrolyte. Region 2 (green) in the Nyquist plot pertains to the intermediate frequency range and is linked to the cathodic reduction of oxygen. On the other hand, region 3 (yellow) corresponds to the low frequency range in the Nyquist plot and is associated with the processes of gas diffusion and transport [48,49,56,57]. The open concept design of the SOFC stack, where the product gas is burned in the afterburner chamber without control over the air streams, and the lack of detailed information about the composition of the product gases, make region 2 less significant for the purposes of this study, and the following EIS analysis will concentrate on regions 1 and 3. Depending on which process is associated with which peak, increasing or decreasing the peak area may or may not improve the SOFC's performance. A rise in region 1 associated with diffusion and gas transport processes, for instance, would indicate greater diffusion resistances. Consequently, this would result in a decline in the SOFC's performance.

### 3.3 Variation of volumetric flow rate and cell temperature

**Fig. 4:**
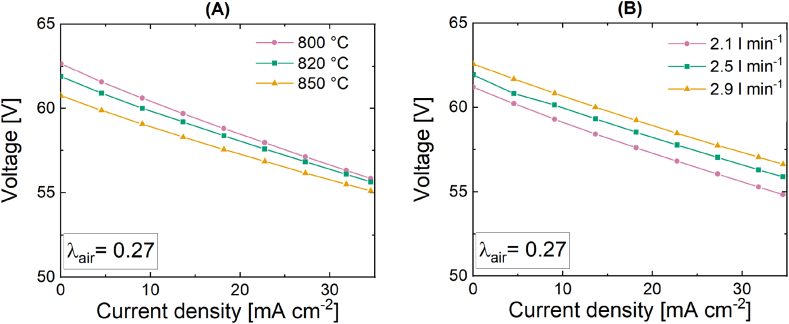
Current-voltage characteristic curves of the SOFC stack under the experimental condition of test 1 (A) variation of the temperature under the constant pre-reformed NG flow of 2.5 l min^−1^ at 800 °C, 820 °C, 850 °C and test 2 (B) variation of the volumetric flow rate with 2.1 l min^−1^, 2.5 l min^−1^ and 2.9 l min^−1^ at a constant temperature of 820 °C.

The outcomes of the first experimental set are depicted in Fig. 4. The graph shown in Fig. 4 (A) showcases the polarization curve corresponding to test 1a, indicating a notable enhancement in system performance at lower temperatures. This result may appear unexpected at first due to the fact that higher temperatures increase both the reaction rates at the electrodes and the ionic conduction of the electrolyte. Furthermore, the low ionic conduction of the electrolyte accounts for the majority of SOFC losses; consequently, the overall performance of the cell should improve as the temperature rises. Conversely, under low loads, such as the present scenario, the impact of the theoretical voltage U_0_, which can be calculated using the Nernst equation and is inversely proportional to temperature, takes precedence over other factors in determining performance [22,45,51]. At a temperature of 800 °C, it can be observed that the voltage attains its maximum value. With an increase in load, the impact of ionic conduction and reaction kinetics becomes more significant. It is believed that the U/i curves for varying operating temperatures will converge at higher loads, and that the higher temperature curve will demonstrate an improvement in stack operation performance. At a current density of 35 mA cm^−2^, it is possible to observe the beginning of this trend. Regarding the impact of the inlet volumetric flow rate (test 1b), it is evident from Fig. 4 (B) that a reduction in the volumetric flow rate leads to a decrease in the performance of the SOFC stack. This result can be understood by examining the definition of electrical efficiency in a SOFC system and the direct relationship between the fuel flow rate and the product of voltage and current. According to existing literature, it has been established that an excessively low volumetric flow rate has the potential to lead to fuel starvation, in the worst-case scenario [26]. It is worth noting that the open circuit voltages (OCVs) exhibit variations in response to changes in the volumetric flow rate, with values ranging from approximately 61 V (at a flow rate of 2.1 l min^−1^) to 62.5 V (at a flow rate of 2.9 l min^−1^). At first glance, it may seem that these results are inconsistent with the Nernst equation, as it is clearly unaffected by the volumetric flow rate. This could be attributed to the natural gas pre-reformation step occurring in the CPOx unit. As a result of varying volumetric flow rate, different gas concentrations exit the CPOx unit, resulting in different equilibrium compositions and subsequently varying OCV values.

**Fig. 5:**
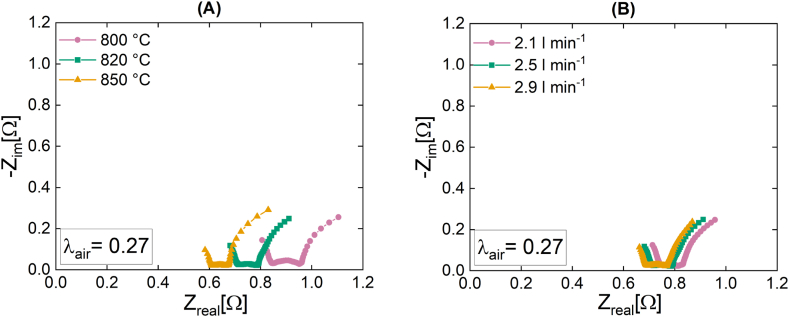
Nyquist plots of the SOFC stack at OCV with a variation of **(A)** the temperature at 800 °C, 820 °C, 850 °C (tests 1a-c) under the constant pre-reformed NG flow of 2.5 l min^−1^ and of **(B)** the volumetric flow rate of 2.1 l min^−1^, 2.5 l min^−1^ and 2.9 l min^−1^ under a constant temperature of 820 °C (tests 2a-c).

The Nyquist plots illustrating the variations in the temperature and the volumetric flow rate of the SOFC stack are presented in Fig. 5. Due to the decrease in overall losses at higher temperatures, a rise in cell temperature (Fig. 5 (A)) results in a noticeable shift to the left of the graph. In particular, an increase in the temperature results in an increase in the conductivity of the electrode and the electrolyte, resulting in lower ohmic resistance. A variation in volumetric flow rate (Fig. 5(B)) results in a change in the impedance plot that is less noticeable. This can be explained by the fact that the variation in volumetric flow has a large effect on gas diffusion processes but no effect on other types of losses, such as polarization or ohmic losses.

**Fig. 6:**
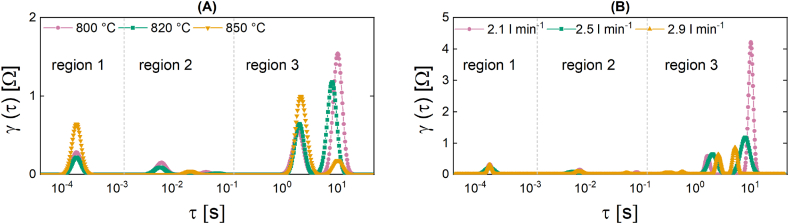
DRT plots of the SOFC stack at OCV with a variation of **(A)** the temperature under the constant pre-reformed NG flow of 2.5 l min^−1^ at 800 °C, 820 °C, 850 °C (tests 1a-c) **(B)** the volumetric flow rate of 2.1 l min^−1^, 2.5 l min^−1^ and 2.9 l min^−1^ at a constant temperature of 820 °C (tests 2a-c).

Comparing the peak area of the regions depicted in the DRT plots in Fig. 6 with the values reported in Table 5 provides additional insight into the processes taking place within the stack as its temperature (Fig. 6 (A)) and volumetric flow rate (Fig. 6 (B)) increase.

**Table 5:** Area underneath the respective DRT plot, according to each region, for the different tests from experimental set 1.

Test no.	Parameter Variation	Peak area region 1	Peak area region 2	Peak area region 3	Total area
		[−]	[−]	[−]	[−]
**1a**	T = 800 °C	1.87 ·10^−5^	8.64 ·10^−4^	6.19	6.1894
**1b**	T = 820 °C	1.44 ·10^−5^	6.70 ·10^−4^	5.31	5.3072
**1c**	T = 850 °C	4.27 ·10^−5^	3.72 ·10^−4^	5.11	5.1131
**2a**	${\dot{V}}_{in}$ = 2.1 l min^−1^	1.09 ·10^−5^	1.52 ·10^−3^	8.28	8.2803
**2b**	${\dot{V}}_{in}$ = 2.5 l min^−1^	1.44 ·10^−5^	6.70 ·10^−4^	5.31	5.3076
**2c**	${\dot{V}}_{in}$ = 2.9 l min^−1^	1.06 ·10^−5^	6.90 ·10^−4^	1.20	1.2026

Since the temperature and gas flow rate changes occurred during an open-circuit-voltage (OCV) operation, it is not expected that the resistances in region 1, which are related to anodic oxidation and ionic conduction processes, will change significantly as the temperature and volumetric flow rate increases. Nevertheless, it is evident that as the temperature rises in region 1, the peak area significantly increases from 1.87·10^−5^ to 4.27· 10^−5^. On the basis of the current set of measurements, it is reasonable to assume that this variation in region 1 corresponds to an increase in ionic conduction. Regarding the variation in volumetric flow rate, there is no discernible change. This behavior appears consistent with the aforementioned Nyquist plot shift. However, it is apparent based on the observed peak area in region 3 that alterations in the fuel flow rate have a substantial impact on the processes of gas transport and diffusion, whereas the influence of temperature is relatively minor. Specifically, altering the temperature of the cell from 800 °C to 850 °C results in a reduction of ∼17 % (from 6.19 to 5.11) in the area of the peak region 3. When the volumetric flow rate of inlet gas is increased from 2.1 l min^−1^ to 2.9 l min^−1^, there is a sharp decline in the region 3 peak, with a reduction of 85.48 % (from 8.28 to 1.20) which can be attributed to a reduction in the resistance associated with gas transport and diffusion losses.

### 3.4 Variation of H/C ratio and coupling of wood gasification

**Fig. 7:**
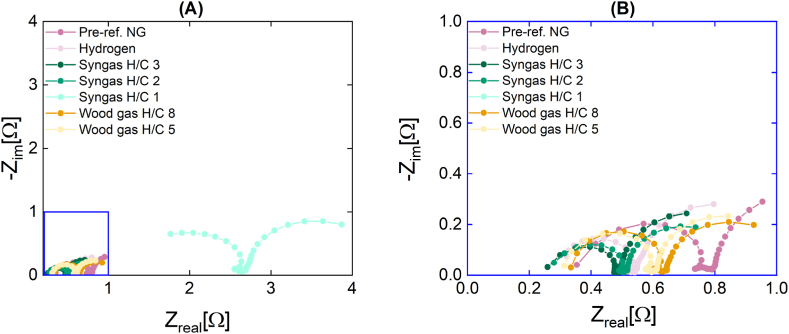
**(A)** Nyquist plot of the SOFC stack and **(B)** a zoomed view of the low impedance area of the same Nyquist plot (right) for real (orange curves) and synthetic wood gas (green curves). Also included as references are the hydrogen and pre-reformed NG curves (pink curves). Operating conditions: T = 820 °C and i_L_ = 10 mA cm^−2^. (For interpretation of the references to colour in this figure legend, the reader is referred to the Web version of this article).

The results of each test conducted during the experimental set have been incorporated into a single Nyquist diagram illustrated in Fig. 7. As can be seen in Fig. 7 (B), which is the enlarged version of the low impedance region of Fig. 7 (A), the Nyquist plots derived from reference measurements conducted with hydrogen and pre-reformed NG (tests 3a and 3b) exhibit a spatial proximity to the Nyquist plots obtained when utilizing syngas (tests 4a-c) and real wood gas (tests 5a and 5b) as the anodic inlet gas. As the H/C ratio decreases from 8 to 1 during syngas operation, the Nyquist plots shift to the right, indicating an increase in the SOFC stack's impedance. The Nyquist plot shows a significant shift between H/C ratios 2 and 1, suggesting a critical operating mixture point where the performance of the SOFC system decreases significantly. The decrease in performance could be due to carbon deposition on the anode surface, causing higher resistance in the stack.

**Fig. 8:**
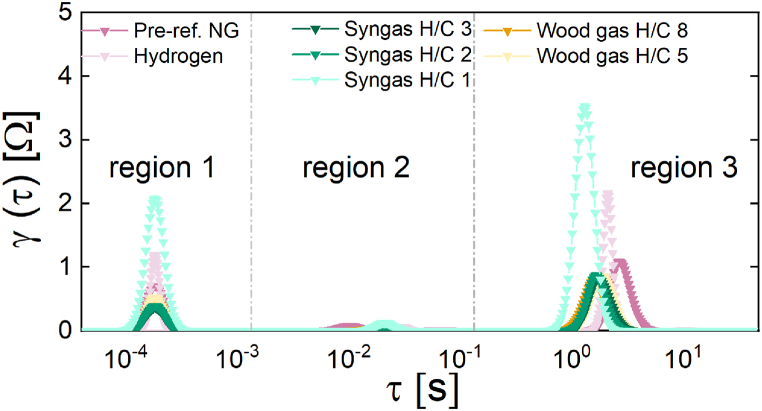
DRT plot of the SOFC stack for real (orange curves) and synthetic wood gas (green curves). Also included as references are the hydrogen and pre-reformed NG curves (pink curves). Operating conditions: T = 820 °C and i_L_ = 10 mA cm^−2^. (For interpretation of the references to colour in this figure legend, the reader is referred to the Web version of this article).

Examining the DRT plots shown in Fig. 8 and the values corresponding to the peak region areas reported in Table 6 allows for a more in-depth analysis of the results of this second experimental set. Since the set was conducted with a load current of 10 mA cm^−2^, in addition to region 3, region 1 will also be considered in the discussion. Upon comparing the peak mathematical area values for both regions, it becomes evident that an increase in the H/C ratio results in an enhanced performance of the SOFC stack. As the H/C ratio increases, the peak areas of region 1 of the DRT plot increases, which is attributed to rapid electrochemical processes occurring at the electrodes, such as the anodic electrooxidation reaction and the ionic conduction of the electrolyte. An increase in the peak area within this particular region would indicate a corresponding increase in the fuel oxidation mechanism, resulting in a greater number of electron/ion transfers per unit of fuel gas and, as a result, contributing to an improved anodic performance of the SOFC stack as a whole. The peak area in region 3 of the DRT plot, which is attributed to gas transport and diffusion resistances, decreases significantly as the H/C ratio and, consequently, the hydrogen content of the inlet gas increases, resulting in an improvement in the performance of SOFC stacks. This can be understood by comparing the volumetric sizes of the hydrogen- and carbon-containing constituents of syngas. Due to the fact that hydrogen is a smaller gas component than carbon, decreasing the amount of carbon (= increasing the H/C ratio) leads to the predominance of hydrogen, therefore enabling improved gas mobility and diffusion. It interesting to note that compared to Fig. 3, Fig. 6, Fig. 8 exhibits two peaks in region 3. The observed discrepancies in the number of peaks between Fig. 3, Fig. 6 (measured under OCV conditions without electron transfer, two peaks) and Fig. 8 (measured under load, one peak) may be attributable to the oxidation process. This oxidation process may reduce the diffusion and gas transport resistances in region 3, resulting in a single peak for this region. Due to technical constraints, a deeper interpretation was unable to conduct a more thorough analysis and validate this interpretation, which should be regarded as a hypothesis.

**Table 6:** Peak area underneath the respective DRT plot, according to each region, for the SOFC stack at 820 °C and 10 mA cm^−2^ during the experimental set 2. Operating conditions: T = 820 °C and i_L_ = 10 mA cm^−2^.

Test no.	Parameter Variation	Peak area region 1	Peak area region 2	Peak area region 3	Total area
		[−]	[−]	[−]	[−]
**3a**	H/C ratio = 4	4.68 · 10^−^^5^	5.60 ·10^−4^	1.19	1.1906
**3b**	H/C ratio = ∞	3.86 · 10^−5^	4.21 ·10^−4^	0.83	0.8305
**4a**	H/C ratio = 3	2.42 · 10^−5^	1.54 ·10^−4^	0.61	0.6102
**4b**	H/C ratio = 2	2.61 · 10^−5^	1.70 ·10^−4^	0.54	0.5402
**4c**	H/C ratio = 1	1.38 · 10^−4^	9.80 ·10^−4^	1.69	1.6911
**5a**	H/C ratio = 8	3.35 · 10^−5^	2.16 ·10^−4^	0.51	0.5102
**5b**	H/C ratio = 5	3.47 · 10^−5^	2.72 ·10^−4^	0.70	0.7003

Analysing the reference measurements (tests 3a and 3b), an opposing effect can be observed. As predicted by our previous observation during the OCV operation of the SOFC stack, the trend in region 1 indicates that the anodic oxidation process in the SOFC system performs better when using pre-reformed NG as the inlet gas rather than hydrogen. In region 3, a different phenomenon is observed: the diffusive resistance of pre-reformed NG is significantly greater than that of hydrogen. This result may be attributable to a reversible carbon deposition at the three phase boundary (TPB) or at the anode surface, which may be caused by a variety of factors, including the accumulation of carbon-containing gases from previous measurements or a possible deactivation of the catalytic activity of the CPOx unit, which results in a less effective reformation of NG. The term “reversible carbon deposition” refers to the complete removal of carbons through the delivery of oxygen, resulting in the restoration of the cell's performance (as depicted in the OCV versus time curve in the supplementary materials section) to its initial state after a predetermined period of time. The carbon deposition reduces gas permeability and makes it particularly challenging for C-containing gases with atoms larger than hydrogen to pass through, resulting in an increased diffusion resistance contribution in region 3.

For an accurate interpretation of the results of the synthetic wood gas experiments (tests 4a-c) pertaining to region 1, the meaning of the H/C ratio must be considered. At a reduced H/C ratio (H/C ratio = 1), there are more C-containing species in the gas, resulting in an increase in the number of electrons that must be transferred pro atom during the oxidation process and, consequently, an increase in the region 1's resistance compared to the case of H/C ratio 3. This could account for the significant rise in resistance observed in region 1 of ∼57 % (from 2.42 ·10^−5^ to 1.38 ·10^−4^). From the analysis of the region 3 it is evident that a critical threshold in the composition of syngas mixtures exists, particularly the mixtures with H/C ratios below 3 with an increase in the overall resistance of ∼36 % (from 1.6911 to 0.6102). Under this threshold, the performance of the SOFC system decreases significantly. On the basis of the Nyquist plot and the DRT analysis, it is clear that the syngas mixture with a H/C ratio of 1 possesses the greatest total resistance, which is quantified with a mathematical area value of 1.69. Consequently, this particular syngas mixture exhibits the lowest overall SOFC-stack performance throughout the test experimental set. Similar to the tests 3a and 3b, this may be the result of a reversible carbon deposition on the anode surface or the TPB. Carbon deposition in this instance may be caused by higher carbon concentrations in the synthetic wood gas, which may have a significant impact on gas permeability and diffusion.

Prior to gaining a comprehensive understanding of the impact of wood gasification coupling on the performance of the SOFC stack, a comparison with the pure hydrogen test is essential. For the first test (5a), in which the inlet gas contained a small amount of real wood gas (2 l min^−1^) diluted with hydrogen, it is evident from the peak area of region 3 only a minor change from the hydrogen to the real wood gas test. A major impact is evident in the peak area of region 1, where a decrease of 38.5 % is observed during wood gas operation (from 0.83 to 0.51). Without additional measurements designed to address this objective, it is difficult to determine the relationship between the variability of diffusion processes and the presence of carbon-based gases and tar constituents in wood gas. When comparing the results of the syngas experiments (tests 4a-c) to those of the real wood gas experiments (tests 5a and 5b), it is expected that an increase in the H/C ratio will reduce the overall resistance, similar to what was observed in the syngas experiments. For the first test (5a) an increase in the H/C ratio from 3 to 8 decreases the molar carbon content by ∼58 % (from 0.07 mol min^−1^ to 0.03 mol min^−1^). In line with the initial assumption, it is observed that the overall resistance decreases by about 16 % (from 0.6102 to 0.5102), but to a lesser extent than expected. During the second test (5b), an increase in the H/C ratio from 3 to 5 reduced the molar carbon content by ∼29 % (from 0.07 mol min^−1^ to 0.05 mol min^−1^). The overall resistance has increased by approximately 14 % (from 0.6102 to 0.7003), in contrast to the initial assumption.

The contrasting results observed during real wood gas tests can be primarily attributed to the increased concentration of tars, specifically naphthalene (150 mg Nm^−3^) and benzene (44.7 mg Nm^−3^), in the inlet gas as the volumetric flow rate of wood gas increases. These compounds have an opposing impact on the SOFC stack's performance. At a certain concentration (60 mg Nm^−3^) in the syngas mixture, naphthalene negatively impacts the performance of SOFCs by preventing the direct reforming of other carbons, such as methane, as well as increasing the diffusion resistance and inhibiting charge transfer reactions [25,27,29,38].

As the concentration of wood gas rises, it could be assumed that the increase in diffusion resistance depicted in region 3 is the primary effect of naphthalene. In addition, some authors suggested that the effect of naphthalene on a SOFC system is dependent on the methane content of the wood gas. When methane levels are high and the SOFC system is subjected to high load current, the naphthalene's influence is more evident [28,29,33]. The low methane concentration in real wood gas generated by the allothermal gasifier, when combined with hydrogen dilution and a low external load current, suggests that the potential negative effects of naphthalene on the performance of the SOFC stack may be quite minor. In contrast, previous studies have demonstrated that the introduction of benzene in quantities of up to 15,000 mg Nm^−3^ over a 24-h period did not yield any discernible impact on the electrochemical functionality of the SOFC [70]. Furthermore, there was an observed increase in the voltage. The introduction of benzene resulted in a notable enhancement in the flow rates of hydrogen and carbon monoxide, whereas the flow rates of carbon dioxide and methane experienced only slight increments [25,30]. However, as the concentration of wood gas in the inlet gas increases, there is only a slight increase of ∼3.5 % in the peak area observed in region 1 of the DRT plot related to the anodic electrooxidation process (from 3.35 ·10^−5^ to 3.47 ·10^−5^) that could be related to the presence of benzene in the wood gas.

## 4 Conclusions

This paper focuses on the operation of synthetic and natural wood gas on a commercially available SOFC stack (from Hexis Galileo 1000 N) using EIS and subsequent DRT analysis. Typically, the DRT spectrum is divided into a number of regions that correspond to the physical processes occurring within the SOFC system. Typically, the DRT spectrum is divided into a number of regions that correspond to the physical processes occurring within the SOFC system. The first region (region 1) focuses on faster processes at the electrodes, including the anode electrooxidation reaction and the ionic conduction of the electrolyte. The second region (region 2) focuses on oxygen reduction at the cathode, whereas the third region (region 3) is concerned with gas diffusion and transport processes.

Using pre-reformed NG as fuel, a first test's experimental set was conducted to evaluate the impact of increasing temperature (tests 1a-c) and varying the amount of anodic inlet gas (tests 2a-c) on the performance of the SOFC stack. A greater amount of fuel reduces the gaseous diffusion and transport resistances, which leads to a reduction of ∼85 % of the peak area corresponding to region 3 of the DRT plots. Due to the decrease in overall losses at higher temperatures, a rise in cell temperature results also in a noticeable reduction in diffusion resistances of ∼75 %. Given that this experimental set occurred in the context of an OCV operation, it can be observed that the contributions in the peak areas of region 1, which are primarily associated with anodic oxidation processes, exhibited minimal changes.

A second experimental set was conducted to investigate the physical processes occurring within the SOFC stack and to assess the effects of integrating wood gasification into the system. Based on the type of fuel gas used to power the stack and the H/C ratio of the inlet gas mixture, this experimental set was divided into three test sections: reference gases (tests 3a and 3b), synthetic wood gas (tests 4a-c), real wood gas (tests 5a and 5b). The reference gas measurements indicate that the anodic oxidation process in the SOFC system performs better when using pre-reformed NG as the inlet gas rather than hydrogen. Furthermore, the diffusion resistance depicted in region 3 for the pre-reformed NG is significantly higher than that of hydrogen. This outcome may be attributable to a reversible carbon deposition at the TPB or anode surface, or a deactivation of the CPOx unit. As the H/C ratio decreases from 8 to 1, an increase in syngas and real wood gas operation causes the Nyquist plots to shift to the right, indicating a rise in the impedance of the SOFC system. Syngas mixtures with H/C ratios below 2 appear to have a critical operating mixture point below which the SOFC system performance decreases significantly; this may be due to a reversible carbon deposition resulting from higher carbon concentrations (C-content for H/C = 1 0.29 mol min^−1^) at the TBP or anode surface. The increase of the content of real wood gas in the inlet gas resulted in contrasting performance trends which were primarily attributed to the increased concentration of tars (from SPE analysis: 150 mg Nm^−3^ for naphthalene and 44.7 mg Nm^−3^ for benzene), which have an opposing impact on the SOFC stack's performance. As the concentration of wood gas in the inlet gas rises, the peak area observed in region 1 of the DRT plot increases by only 3.5 % which can be attributed to the presence of benzene in the gas mixture. In region 3, naphthalene had a more significant impact, resulting in an increase in diffusion resistance. The influence of tars on the operational efficiency of SOFC stacks appears to be mitigated by their low concentrations and subsequent dilution with hydrogen, along with their opposing effects. The aforementioned phenomenon results in negligible changes to the anodic oxidation process occurring within the SOFC stack.

Future measurements should focus on enhancing the tar concentration in the inlet gas that enters the SOFC system. This will allow for a more comprehensive analysis of the influence of specific tars on the electrochemical reactions within the system. Additionally, by combining EIS and DRT analysis, it will be possible to identify further constraints associated with the integration of a SOFC system with wood gasification.

Based on the important main findings in this paper, several recommended integration strategies for biomass gasifier-SOFC systems can be identified. Allothermal gasifiers operating at high temperatures (around 800 °C) and low pressures (0.6 bar) coupled with SOFC systems offer promising integration strategies. These gasifiers should be capable of providing a volumetric flow rate suitable for SOFC operation while ensuring optimal gas composition. Additionally, utilizing pre-cleaning units, such as scrubbing bottles and active carbon filters, to remove tars and sulfur components from the gas before entering the SOFC stack is crucial. Furthermore, upstream desulfurization systems are recommended to prevent degradation issues. Maintaining operating temperatures within the optimal range, typically between 750 °C and 850 °C, is essential for maximizing system efficiency and fuel utilization. Moreover, ensuring a H/C ratio greater than 2 in the inlet gas mixture is necessary to avoid performance losses and material degradation.

Additionally, scaling up biomass gasifier-SOFC systems to commercial scales presents several challenges and limitations. Technical challenges, such as transient signal noises during electrochemical analysis and catalytic activity decline over time, need to be addressed to ensure reliable and consistent system performance. Achieving commercial viability requires overcoming challenges related to system integration, component durability, and cost-effectiveness. Identifying suitable applications for biomass gasifier-SOFC systems is crucial. Applications requiring high overall energy efficiency, low environmental impact, and reliable power generation are the most suitable candidates. In conclusion, the integration of biomass gasification with SOFC systems presents a promising pathway for high-efficiency power generation. However, addressing the identified integration strategies and overcoming future challenges are essential for realizing the full potential of these systems in commercial applications.

## Data availability statement

The authors confirm that the data supporting the findings of this study are available within the article [and/or] its supplementary materials.

## CRediT authorship contribution statement

**Federica Torrigino:** Writing – original draft, Visualization, Validation, Methodology, Investigation, Formal analysis, Conceptualization. **Fabian Grimm:** Writing – review & editing, Methodology, Investigation, Conceptualization. **JÜrgen Karl:** Writing – review & editing, Project administration, Funding acquisition. **Katharina Herkendell:** Writing – review & editing, Supervision.

## Declaration of competing interest

The authors declare that they have no known competing financial interests or personal relationships that could have appeared to influence the work reported in this paper.
