# Supplementary material for: In-situ electrochemical impedance analysis of a commercial SOFC stack fueled by real wood gas
Source: Heliyon. 2024 Jun 6;10(12):e32509. doi: 10.1016/j.heliyon.2024.e32509 (PMC11215268; doi:10.1016/j.heliyon.2024.e32509)
Supplement: Multimedia component 1 [file mmc1.docx]

**Supplementary material**

1. **NOVUM DC/DC converter**

The NOVUM DC/DC converter underwent extensive testing to ensure its reliability and efficiency. When testing, the device's performance was assessed based on various important specifications. Firstly, the converter demonstrated its versatility by effectively handling input voltages within the range of 25 V to 65 V, accommodating diverse power supply configurations. Users have the flexibility to customize the input current, ranging from 0.5 A to 4 A, to meet specific application requirements. The converter is designed to maintain a stable output voltage of 75 V, as required by the connected electronic load, ensuring consistent and reliable performance. Additionally, its robustness is verified through its ability to manage a maximum input power of 260 W, guaranteeing dependable operation even in demanding conditions. Furthermore, the device is equipped with a reliable 5 V voltage for both its operation and driver circuitry, providing precise regulation and control. Safety is prioritized with the inclusion of a quick-acting 5 A fuse in the input circuitry, protecting against overcurrent scenarios. Moreover, the communication interface is facilitated through an RS485 interface, following the Modbus protocol. This enables seamless communication with the converter for monitoring, control, and data retrieval purposes, ensuring integration into larger systems or networks.


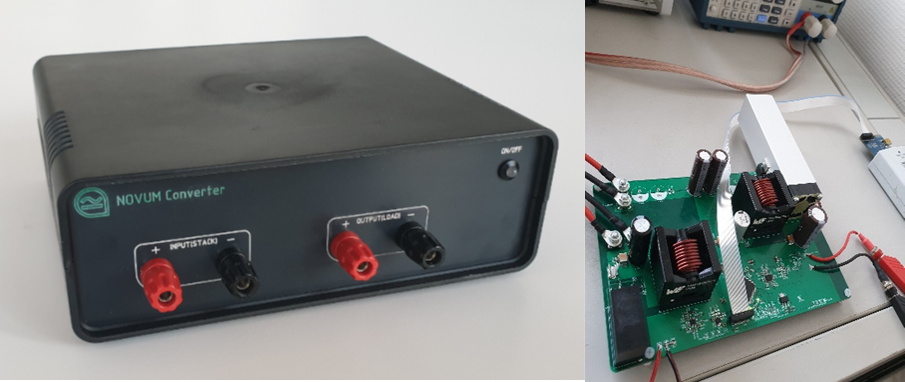


Figure 1: External view and internal components of of the NOVUM DC/DC Transformer.

1. **Validation of the EIS spectra**

Prior to commencing the experimental test in the SOFC stack, it was crucial to check the reliability of the EIS spectra to ensure that the DC-DC converter did not impact the EIS spectra of the stack during its operation. For this purpose, a RC circuit was set up using a 1000 μF electrolytic capacitor and a 0.1 Ω resistor to simulate the impedance of the stack. EIS tests were conducted on the system by gradually increasing the applied DC voltage to the circuit up to 65 V, with and without the DC-DC converter. The utilized EIS-parameters are shown in Table 1.

Table 1: Parameter utilized for the EIS-test of a RC-circuit (R=0,1 Ω; C=1000 µF).

| **Start frequency** | **Stop frequency** | **Offset** | **Amplitude** |
| --- | --- | --- | --- |
| *[Hz]* | *[Hz]* | *[A]* | *[A]* |
| 1 | 3500 | 0,15 | 0,1 |

In Fig. 2 is possible to see the EIS spectra of the RC circuit with and without DC-DC converter.


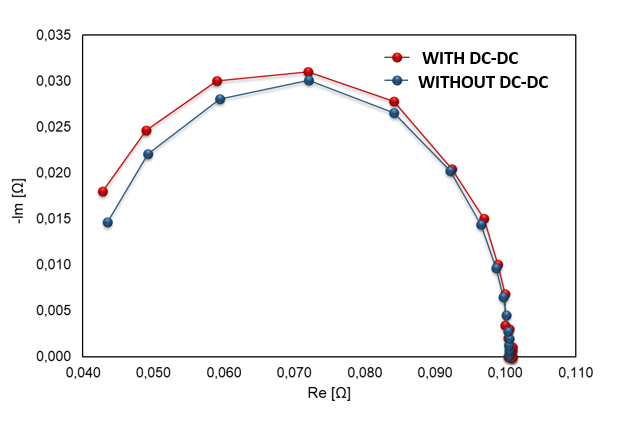


Figure 2 EIS Spectra Comparison of RC Circuit with and without DC-DC Converter. The EIS spectra of the RC circuit, simulating the stack impedance, are depicted under two conditions: with and without the presence of the DC-DC converter. Applied DC-potential from the source: 60 V.

The integration of the DC-DC converter has a minimal impact on the measured impedance spectra. Considering the slight differences in the test setups, these minor deviations can be neglected. While acknowledging the limitations of this approach, the authors emphasize the importance of mitigating risks and ensuring the integrity of the data. Despite not directly measuring the impedance characteristics of the DC-DC converter stack side, the chosen methodology provided valuable insights into the device's behavior and its impact on impedance spectra measurements. Additionally, the authors highlight the significance of the DC-DC converter in the measurement loop. Signal noises observed in previous campaigns under load were attributed to transient components on the regulated current of the electronic load (I_el_). Integrating the DC-DC converter aimed to isolate EIS from these interferences, effectively cleansing the SOFC stack's impedance (Z_stack_). With the addition of the DC-DC converter the potential drop across the internal resistance of the SOFC stack (Uz) is dependent on the set constant input current of the DC-DC converter(I_L_) and not anymore from the electronic load current. The DC current from the converter produces a DC voltage across the internal resistance, which is isolated from the measurement signal by the AC component. The difference of the setup without converter(a) and witch converter(b) are shown in Fig. 3.


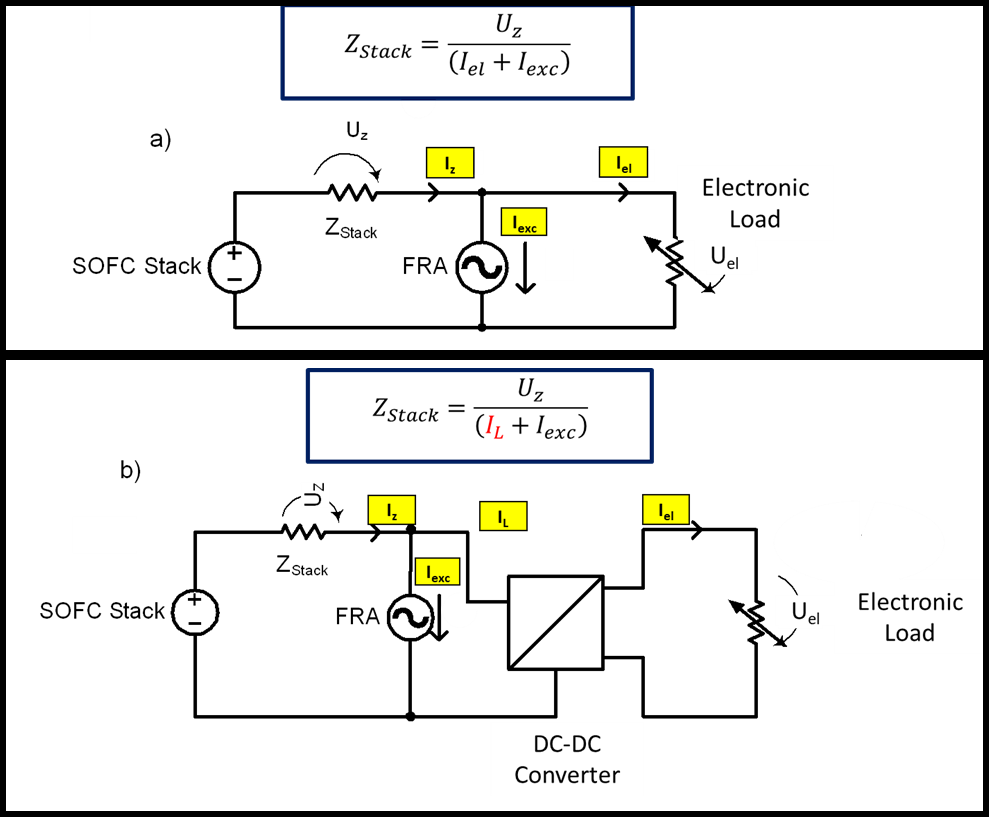


Figure 3 Comparison of the two measurement-loop circuits setups: (a) without the DC-DC converter and (b) with the DC-DC converter integrated. The addition of the DC-DC converter influences the potential drop across the internal resistance of the SOFC stack (U_z_), as it is dependent on the set constant input current of the converter (IL). The DC current from the converter generates a DC voltage across the internal resistance, which remains isolated from the measurement signal by the AC component.
